# Impact of Marine Drugs on Cytoskeleton-Mediated Reproductive Events

**DOI:** 10.3390/md8040881

**Published:** 2010-03-25

**Authors:** Francesco Silvestre, Elisabetta Tosti

**Affiliations:** 1 Institute of Food Sciences, National Research Council, via Roma 52A/C, 83100-Avellino, Italy; E-Mail: fsilvestre@libero.it; 2 Animal Physiology and Evolution Laboratory, Stazione Zoologica Anton Dohrn, Villa Comunale, 80121-Naples, Italy

**Keywords:** marine drugs, toxins, reproduction, microtubules, microfilaments

## Abstract

Marine organisms represent an important source of novel bioactive compounds, often showing unique modes of action. Such drugs may be useful tools to study complex processes such as reproduction; which is characterized by many crucial steps that start at gamete maturation and activation and virtually end at the first developmental stages. During these processes cytoskeletal elements such as microfilaments and microtubules play a key-role. In this review we describe: (i) the involvement of such structures in both cellular and *in vitro* processes; (ii) the toxins that target the cytoskeletal elements and dynamics; (iii) the main steps of reproduction and the marine drugs that interfere with these cytoskeleton-mediated processes. We show that marine drugs, acting on microfilaments and microtubules, exert a wide range of impacts on reproductive events including sperm maturation and motility, oocyte maturation, fertilization, and early embryo development.

## 1. Introduction

Marine organisms represent a huge source of bioactive compounds affecting reproductive processes (for a review see [1]). Among them, marine natural products targeting microtubule or microfilament structures and dynamics are of particular interest, since reproduction is a complex multi-step process in which the cytoskeleton plays a key-role in the regulation of many functions such as: intracellular transport, cellular shape, motion and division. Microfilaments and microtubules are the main components of the cytoskeleton in eukaryotic cells, forming an extensive network; but, despite many studies, there are some aspects still obscure, such as polymer dynamics. In fact, on the basis of the Wegner theory [2], the chemical state of the bound nucleotide determines the rates of subunit addition and removal. More recently, was proposed that “structural plasticity” is the change in the structural state of polymer without change in the chemical state of its bound nucleotide [3]. Thus, integration with the older treadmilling theory to clarify these dynamics should be necessary.

Here, we describe how marine drugs affect cytoskeleton mediated reproductive events by giving an overview on: (i) structure, functions and dynamics of microfilaments and microtubules; (ii) the main reproductive events such as gamete maturation, activation, fertilization and early embryo development; (iii) how cytoskeleton elements are involved in these processes and how marine toxins affect reproductive events throughout the cytoskeletal network. In this review, we show that marine drug studies may help to better understand the cytoskeleton role in reproduction from invertebrates to mammals.

## 2. Microfilaments or Actin Filaments

### 2.1. Nucleation and Function of Microfilaments

The actin cytoskeleton is a dynamic network of filaments made up of a monomeric 43 kDa protein named globular actin (G-actin), which self-assembles into polymers of 8 nm diameter that are also called microfilaments or filamentous actin (F-actin) [4]. In cells, approximately half of the actin is kept in monomeric form, and the polymerization of actin is a dynamic process. Generally speaking, F-actin networks are continuously reorganized in cells that rapidly change their shape and in fast migrating cells that swiftly change the direction of movement [5]. Continuous polymerization and depolymerization of actin molecules in cell-surface protrusions have been well investigated and defined; in fact conversion of these two states of actin existence, which is the foremost point of actin functional performance, is very essential for cell survival [6]. The function of the actin cytoskeleton in cells relies on the intrinsic capacity of the actin monomers to reversibly assemble into protein polymers. Actin is an asymmetric molecule, assembling into polar filaments with structurally and functionally distinct ends, characterized by ATP-actin monomer addition at the plus-end (or barbed-end) and loss of ADP-actin monomers at the minus-end (or pointed-end) [4].

The nucleation and functions of microfilaments have been extensively investigated. It is known that new actin filaments are formed by cutting of existing filaments or *de novo* by the action of specialized nucleating components. One of the most highly characterized nucleating component is the Arp (actin-related protein) 2/3 complex, which catalyzes actin polymerization [7,8]. To maintain a steady state, filaments undergo depolymerization facilitated by actin-depolymerizing factor (ADF)/cofilin to limit the rate of new filament nucleation and elongation [9–11].

Generation of free barbed ends by nucleating units at specific sites, together with release of ATP-monomers by monomer-buffering proteins (thymosin β4, profilin), drives rapid elongation of actin filaments at barbed ends. Subsequently, old filaments are capped by proteins such as gelsolin and CapZ. Hydrolysis of ATP constitutes a timer mechanism for filament turn-over. Cofilin specifically depolymerizes ADP-filaments, increasing the formation of actin monomers. Profilin increases ADP-ATP exchange in the actin monomers released from cofilin, and together with thymosin β4, forms a polymerization-competent ATP monomer pool that will be recycled to free barbed ends [12,13]. It is important that the function of profilin and cofilin is not restricted to cell migration. There are studies suggesting a role for these proteins in the regulation of actin dynamics during the assembly and disassembly of the contractile ring [4,14–16], but also in the modulation of intracellular calcium signaling [17].

### 2.2. Marine Toxins Interfering with Microfilament Network

Besides endogenous actin filament-regulating proteins, numerous natural products also display potent abilities to affect the polymeric state of actin filaments [18,19]. This property has earned these compounds significant recognition as valuable molecular probes for dissecting complex cellular pathways that are dependent upon the actin cytoskeleton [4,18,20].

**Figure 1:**
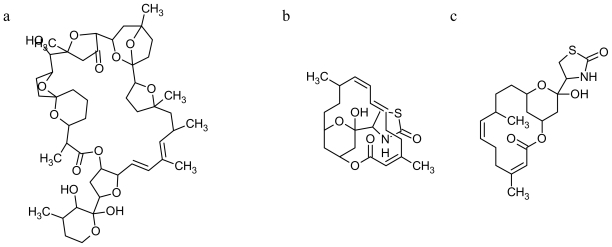

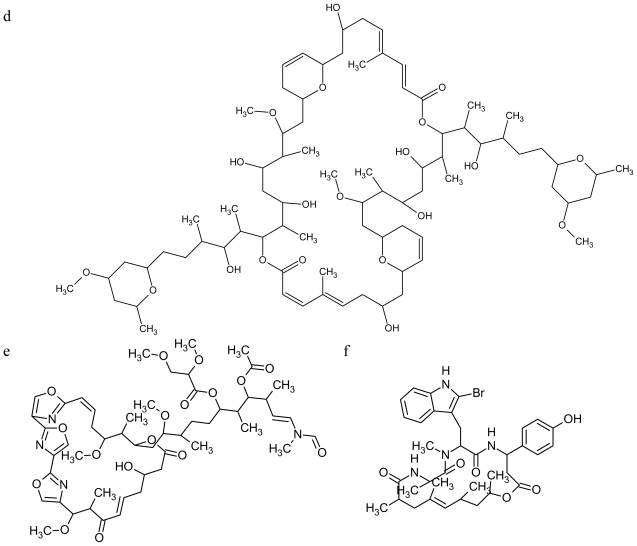
Chemical structures of marine drugs binding actin (from Pubchem website): **PTX-2** (a); **LAT A** (b); **LAT B** (c); **SWI** (d); **MYC** (e); **JAS** (f).

Although they can share a common structure (Figure 1) and target (Table 1), anti-actin drugs display diverse mechanisms of action. Toxins that block or destabilize actin filaments have been shown to act by binding two distinct regions of the actin monomer: (i) the ATP-binding cleft and (ii) the barbed end. Due to the dynamic nature of actin filaments, filament-destabilizing compounds can be further subdivided into those which merely sequester actin monomers when they passively dissociate from filaments, and those that also actively promote filament severing by binding directly to the filament and disrupting interactions between adjacent actin monomers [21].

There is a wide diversity of mechanisms of action for marine toxins, but, despite this complex array of modes of action, only **pectenotoxins** (**PTXs**, Figure 1a) are known to target specifically the actin filaments**. PTXs** mimic the activity of endogenous actin-binding proteins to varying degrees [18]. These toxins were first isolated from the Japanese scallop *Patinopecten yessoensis* [22], but they are produced by many species of the dinoflagellate genus *Dinophysis*. **PTXs** induce actin filament disruption by a capping effect and G-actin monomer sequestration [23].

Cytotoxins **Latrunculin A** and **B** (**LAT**, Figure 1b and 1c, respectively), isolated from the Red Sea sponge *Latrunculia magnifica*, are potent inhibitors of actin filament formation [24,25]. **LATs** specifically sequester monomeric actin, mimicking proteins such as β-thymosins. They inhibit polymerization of G-actin, promote depolymerization of F-actin most likely by an allosteric mechanism [18,25,26], and form ternary complexes with profilin or thymosin β4-actin *in vitro* [27]. They are the only known toxins that interact with the ATP-binding cleft of the actin monomer [21,25–30]. **LAT A** in the nanomolar concentration range disrupts the actin cytoskeleton and causes cell rounding [24,31].

Different classes of natural products target the barbed end instead of the ATP-binding cleft. **Swinholide A** (**SWI**, Figure 1d) is a dimeric macrolide, isolated from the marine sponge *Theonella swinhoei* [21,32]. *In vitro*, **SWI** severs actin filaments without capping them. In cells, **SWI** disrupts the actin cytoskeleton by depolymerizing F-actin, probably due to sequestering actin dimers during the nucleation phase of filament growth. In addition, **SWI** is cytotoxic and inhibits cytokinesis [33]. Due to its ability to mimic actin binding proteins, **SWI** can be used to replace deficient capping or severing proteins [4]. Another natural product targeting the barbed end region is **mycalolide B** (**MYC**, Figure 1e): isolated from the marine sponge *Mycale* sp. as an antifungal or cytotoxic substance [34]. **MYC** does not accelerate actin polymerization, but quickly depolymerizes F-actin [35–37]. It inhibits polymerization of purified actin, apparently by sequestration of monomeric actin and actin severing caused by the rapid F-actin depolymerization [18,25]. **MYC** disrupts actin filaments, inducing cell rounding [4,35]. This toxin is considered a depolymerizing agent, and it has become an important tool for elucidating actin-mediated cellular functions [38].

**Jasplakinolide** (**JAS**, Figure 1f) from the marine sponge *Jaspis johnstoni*, has both fungicidal and antiproliferative activity. This peptide potently induces actin polymerization *in vitro*, stabilizing actin filaments and actin nucleation [39,40]. This drug binds F-actin competitively with phalloidin, but despite this similar affinitiy for F-actin, **JAS** seems to stabilize filaments more effectively. **JAS** is able to penetrate cells, thereby representing an extremely useful tool to study actin-dependent processes in cells. Thymosin β4 amplifies the *in vitro* effects of **JAS**, suggesting cellular variations in the concentration of this actin-binding protein may modulate the effects of the drug [40].

However, regardless of the mechanism of action, most of the toxin groups have profound effects on cytoskeletal dynamics [41].

**Figure 2:**
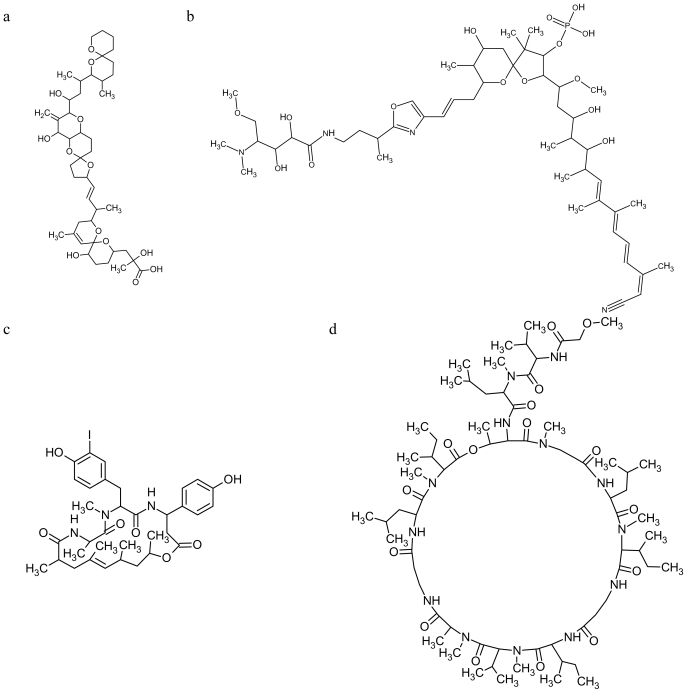

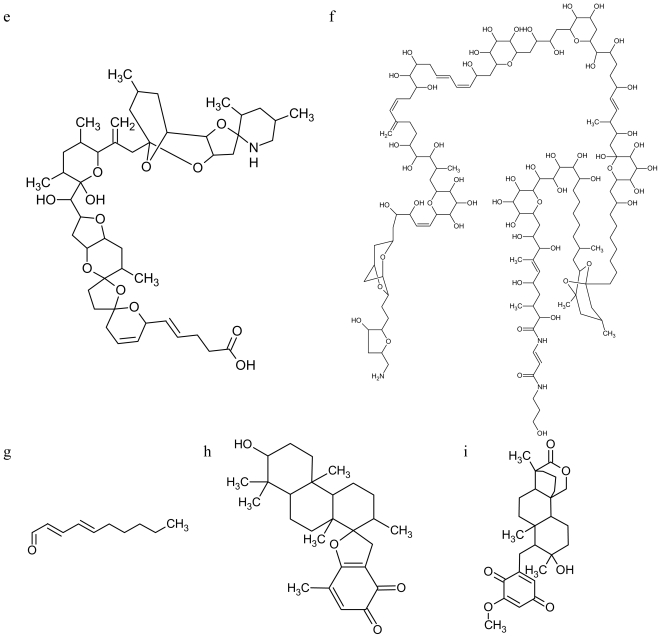
Chemical structures of marine drugs affecting actin dynamics (from Pubchem website, except **PAL** from Chemspider website): **OA** (a); **CLA** (b); **GEO H** (c); **TEO** (d); **AZA-1**(e); **PAL** (f); **DD** (g); **PSE** (h); **STR-26** (i).

Isolated from marine dinoflagellates of the genera *Dinophysis* and *Prorocentrum*, **okadaic acid** (**OA**, Figure 2a) and its analogs, the **dinophysistoxins**, are potent inhibitors of protein phosphatases 1 and 2A [42]. The number of physiological processes in which the Ser/Thr protein phosphatases are involved is immense, including regulation and coordination of the cell cycle [43]. Protein phosphorylation and dephosphorylation events have been established as key factors in the regulation of cytoskeletal structure and function [44]. **OA** has been shown to stimulate cell motility, loss of stabilization of focal adhesions and consequently a loss of cytoskeletal organization; **OA** does not modify the total amount of F-actin, but it causes changes in the F-actin cytoskeleton, with strong cell retraction and rounding, and in many cases cell detachment [42]. It is plausible that **calyculin**-**A** (**CLA**, Figure 2b), originally derived from the marine sponge *Discodermia calyx*, could have the same effect of **OA** on the cytoskeleton, since it is similar to **OA** in its potent inhibition of protein phosphatases 1 and 2A [45]. However, **CLA** differs from **OA** in that protein phosphatases 1 and 2A display greater sensitivity to **CLA** [45,46].

Rangel and colleagues [47] reported new information on the peptides **geodiamolides** (**GEO**) A, B, H and I (Figure 2c) isolated from the marine sponge *Geodia corticostylifera*. These authors noted that peptides A and H had negative effects on proliferation of cancer cell lines by disorganizing F-actin in a dose-dependent manner. Interestingly, normal cell lines did not show cytoskeleton alterations after treatment with **GEO**, thus suggesting a putative biomedical potential for these novel compounds [48]. For the chemical structure see Figure 2c. Isolated from another sponge species, *Petrosia* sp., **theonellapeptolide Ie** (**TEO**, Figure 2d) was shown to have ionophoretic activity and to induce morphological changes of the immature oocytes of starfish *Asterina pectinifera* characterized by disturbed cortical F-actin distribution with assembled dots and rings. However, this action on the maturation appears to be unrelated to the movement of monovalent ions across the cell membrane [49–51].

The **azaspiracids** (**AZAs**, Figure 2e) are a group of marine phycotoxins discovered during the late 1990s. Since then, 20 different analogs of the **AZA** group have been detected in natural samples [52–57]. One of the *in vitro* signs of **AZA** toxicity is the alteration of the actin cytoskeleton arrangement, which is accompanied by changes in cell shape and loss of cell adherence to the substrate, but the biological target of the toxin is still unknown. Moreover, the cytoskeletal damage is irreversible after toxin withdrawal [58].

**Palytoxin** (**PAL**, Figure 2f) is a marine toxin first isolated from zoanthids (genus *Palythoa*), even though dinoflagellates of the genus *Ostreopsis* are the most probable origin of the toxin [59,60]. This marine toxin is known to act on the sodium pump and elicit an increase in sodium permeability, which leads to depolarization and a secondary calcium influx, interfering with some functions of cells. Studies on the cellular cytoskeleton have revealed that the signaling cascade triggered by **PAL** leads to actin filament system distortion. The activity of **PAL** on the actin cytoskeleton is only partially associated with the cytosolic calcium changes; therefore, this ion represents an important factor in altering this structure, but it is not the only cause [61].

Diatoms synthesize bioactive **oxylipins** in response to wound-activation. **2*E*,4*E*-decadienal** (**DD**, Figure 2g) and **decatrienal** are polyunsaturated aldehydes (**PUA**), which are the most intensively researched of the diatom-oxylipin family. Oxylipins are broadly cytotoxic with potential molecular targets associated with the cytoskeleton, calcium signaling and cell death pathways; however, they do not induce the same acute toxicity syndromes as the other algal biotoxins [62], prompting some researchers to question whether or not the oxylipins are indeed toxins in the conventional sense [63]. These defensive compounds are toxic to developmental stages of a range of invertebrate species including copepods, sea urchins, polychaetes and ascidians. Diatom extracts and the bioactive unsaturated short-chain aldehyde **DD** affect microtubule and microfilament stability [64].

**Pseudopterolide** (**PSE**, Figure 2h) is an inhibitor of cell division isolated from the soft coral *Pseudopterogorgia acerosa*. Although its *in vitro* target is unknown, *in vivo* it has been shown to give rise to an aberrant aster formation in sea urchin embryos [4].

The cytoskeleton target of numerous compounds isolated from the marine sponge *Strongylophora* and named as **strongylophorines** (**STR**) is unknown, except **STR-26** (Figure 2i) that affects actin dynamics by inhibiting actin regulator Rho-GTPases [65].

## 3. Microtubules

### 3.1. Microtubule Formation, Functions and Dynamics

Microtubules, the major component of the highly dynamic mitotic spindle, are also key actors in organizing the spatial distribution of organelles in interphase cells, and are extremely stable components of cilia, flagella, and the centrioles.

Microtubules are hollow tubes about 25 nm in diameter with walls made from globular protein α and β-tubulin heterodimers (100 kDa) that associate to form protofilaments running lengthwise along the wall with the α-tubulin subunit facing the microtubule minus end and the β-tubulin subunit facing the plus end. This confers them a structural polarity that is an essential feature. Microtubule assembly is accompanied by hydrolysis of GTP associated with β-tubulin so that microtubules consist principally of ‘GDP-tubulin’ stabilized at the plus end by a short ‘cap’. The polar nature of the microtubule polymer and the hydrolysis of GTP that occurs during microtubule polymerization creates two unusual forms of dynamic behavior in cells and *in vitro*. One such form is dynamic instability [66], in which microtubule ends stochastically switch between episodes of prolonged growth and shortening. One microtubule end, the plus end, shows more dynamic instability behavior than the opposite or minus end. The other form of dynamic behavior, treadmilling, which is due to differences in the critical subunit concentrations at opposite microtubule ends [67,68], consists of net growing at the microtubule plus ends and net shortening at the minus ends [20]. Actin filament treadmilling but not dynamic instability has been observed *in vitro* and in cells [69,70]. For review see [71,72].

In the cell, microtubules are nucleated preferentially in a region called the centrosome or MTOC and grow outwards toward the cell membrane with the plus end leading [73].

Microtubules are important in the process of cell division, e.g., mitosis and meiosis. In mitosis, the microtubules radiating from the centrosome are in a state of dynamic instability and with a number of lengthening and shortening cycles (search-capture model): just the microtubule reaching the kinetochore is stabilized by its interaction with this structure. After correct positioning, each aster forms a spindle body. As mitosis proceeds, each copy of a duplicated chromosome is separated and assembled by the centrosome to which it is attached [30]. Correct spindle assembly is one the cell cycle checkpoints [74,75]; disturbance of spindle formation with inhibitor of microtubule function arrests the cell cycle at mitosis [30].

A large number of proteins interact with microtubules including the motor proteins kinesin and dynein, which use ATP-derived energy to transport a variety of intracellular cargoes toward the plus-ends and minus-ends of microtubules, respectively [76,77]. Kinesin and dynein can take many consecutive steps along their microtubule tracks without dissociating [78–84], allowing them to shuttle cargoes over long distances spanning between a cell center and periphery which use microtubules as pathways for transport; in addition, they are also involved in cell division [85]. Thus, if microtubules are the railway for particle transport within the cell, kinesin and dynein are thought to function as vehicles that carry different cargoes along microtubules [86]. Cytoplasmic dynein is a microtubule-associated motor protein complex with 1000–2000 kDa (for review, see [87]). It is known to generate the minus-end-directed movement along the microtubules (for reviews, see [87,88]).

### 3.2. Marine Toxins Interfering with Microtubules

In spite of their involvement in reproductive processes and unlike microfilament interfering drugs, so far few marine natural products affecting microtubules have been described.

**OA** and **dinophysistoxin-1** (35S-methylokadaic acid) cause rapid changes in the structural organization of intermediate filaments, followed by a loss of microtubules, solubilization of intermediate filament proteins, and disruption of desmosomes [42]. In mouse oocytes, protein phosphatases 1 and/or 2A are positively involved in the activation of pericentriolar material into microtubule organizing centres (MTOCs). This explains the inhibitory effect of **OA** on spindle assembly [89].

**Figure 3:**
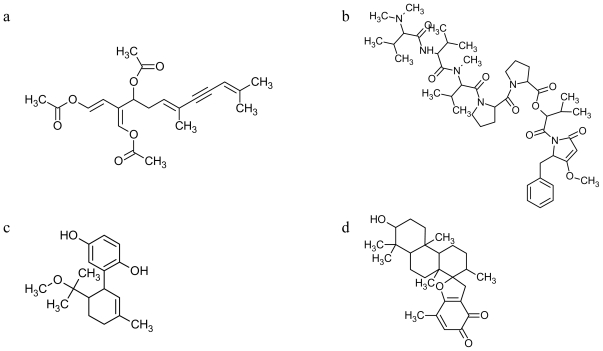
Chemical structure of marine toxins interfering with microtubules: **CYN** (a); **DOL-15**(b); **MET**(c); **STY** (d).

Species of *Caulerpa* were among the first green algae that were investigated by natural product chemists [90]. Extracts of *Caulerpa* spp. and **caulerpenyne** (**CYN**, Figure 3a) have been reported to show diverse biological activities, such as antimicrobial [91–94], antiproliferative [91,95,96] and neurological [90,97]. Electron microscopy analysis indicated that **CYN** treatment results in inhibition of microtubule polymerization probably due to the induced aggregation of tubulin [98].

The shell-less mollusc *Dolabella auricularia* has yielded a number of cytotoxic peptides and depsipeptides (for a review, see [99]). The most potent of these compounds have been dolastatins 10 and 15 [100,101], both of which interact with tubulin and arrest cells in mitosis. **Dolastatins 15** (**DOL**, Figure 3b) is a tubulin destabilizing drug that inhibits microtubule assembly through a vinca-alkaloid domain [102].

Marine toxins have been isolated even from chordates: **methoxyconidiol** (**MET**, Figure 3c) was extracted from the ascidian *Aplidium aff. densum* [103] and most likely it is able to affect microtubule dynamics [104].

**Stypoldione** (**STY**, Figure 3d) from alga S*typopodium zonale* was found in early studies to inhibit polymerization of tubulin into microtubules *in vitro*, which leads to the suggestion that inhibition of microtubule polymerization in cells might be responsible for the ability of the compound to inhibit cell division [105]; however, White and Jacobs [106] showed that **STY** does not act like a mitotic spindle poison. A few years later, **STY** was found to react covalently with the sulfhydryl groups of a number of proteins including tubulin and with sulfhydryl groups of peptides and small molecules. Thus, **STY** could potentially react with a large number of cellular targets and, although it can disrupt microtubules at relatively higher concentrations, it inhibits cell division at the lowest effective concentrations by a selective action on cytokinesis through a mechanism that does not appear to involve disassembly of microtubules [107].

## 4. Reproduction

Reproduction is the biological process by which new individuals are generated. In sexual reproduction, the newly created organism has a combination of half of the genetic material of each parent through the production of the two gametes: spermatozoon and oocyte. The two gametes are formed during peculiar processes such as spermatogenesis and oogenesis, both characterized by meiosis - the unique process of cell division occurring only in gametes, whose goal is the production of haploid cells highly specialized for fertilization.

The main steps toward fertilization are gametogenesis (spermatogenesis and oogenesis), gamete reciprocal activation, sperm-egg interaction, syngamy (fertilization) that leads to the zygote formation. After that, mitotic division of the zygote triggers embryo development

### 4.1. Effect of Marine Drugs on Cytoskeletal Dynamics Involved in Reproductive Events

#### 4.1.1. Spermatogenesis

Spermatogenesis is the process of sperm production from a primordial germ cell, which goes through a highly orchestrated series of stages of generating spermatogonium, primary spermatocyte, secondary spermatocyte, spermatid, and finally mature spermatozoon.

Sperm maturation is defined as the acquired ability of spermatozoa to fertilize eggs. In this process, the sperm undergoes morphological, biochemical, and physiological modifications initially in the testis (testicular maturation) and later in the epididymis in mammals. In the former, maturation occurs at molecular levels especially during the last phase of spermatogenesis known as spermiogenesis; here, the large round haploid spermatid undergoes dramatic morphological and molecular changes including replacement of histones with protamines, high condensation of chromatin, formation of the acrosome, centrioles migration, and tail assemblage. In the meantime, the sperm acquires a functional competence, e.g., acquisition of flagellar beating to provide forward propulsion and compactness of nuclear and flagellar structures [108,109].

Subsequently, in mammals, full sperm functionality occurs in the epididymis, whereas in marine animals it takes place when spermatozoa are spawned in the sea water [108,110,111].

This peculiar developmental process continues throughout nearly the whole lifetime of animals. During spermatogenesis, the above-mentioned structural and biochemical changes that take place in the testis [112], and its gradual differentiation are thus heavily dependent on the cytoskeletal organization. In light of recent data, it has been shown that actin cytoskeleton dynamics play an indispensable role in facilitating these events [6].

In the spermatogenetic process, developing germ cells of different phases migrate from the basal through the intermediate to the adluminal compartment of the testis, *via* junctional contacts and the cytoskeletal apparatus to form round, elongating, and elongated spermatids.

In mammalian testis, the actin-based Sertoli ectoplasmic specialization (ES), which is known as basal ES between Sertoli cells as well as apical ES between Sertoli and developing sperm cells [113], is a specialized type of the adherens junctions in the seminiferous epithelium. ES is the best-characterized cell-to-cell anchoring junction type using an actin filament attachment site in the testis [114,115]. Cheng *et al.* [116] discovered that disruption of Sertoli-germ cell adhesion function by adjudin (AF-2364) in the rat testis can be limited to apical ES without affecting the other junctions, e.g., desmosome-like junctions at the blood-testis barrier site. However, this phenomenon could be a novel approach for male contraceptive development, without the potential side-effects of a drug based on altering the balance of sex hormones, with trials on laboratory animals showing that the contraceptive effect is reversible and that there are no apparent long-term side-effects [6].

Dynein and myosin are found in the apical ES and these proteins apparently function as transporters to assist spermatid translocation across the seminiferous epithelium during spermatogenesis [117–119]. In essence, motor proteins use microtubules found in Sertoli cells at the apical ES site as a track to facilitate the movement of elongating/elongated spermatids across the epithelium [117,119–121]. Cytoplasmic dynein associates with the cytoplasmic face of ER at the site of ES, co-localizing with actin [120,122–124].

Forces on kinetochore microtubules applied by actomyosin could promote tubulin flux of kinetochore microtubules in metaphase-I crane-fly spermatocytes: kinetochore microtubule flux disappeared when cells were treated with an antitubulin drug which decreases microtubule dynamics; moreover, the flux disappeared when cells were treated with anti-actin drugs (**LAT B** and **SWI**), but not when treated with the actin stabilizing drug **JAS**. After treatment with **LAT B** and **SWI**, also spindle actin was altered. These results suggest that actomyosin could be involved in driving the flux of tubulin in kinetochore microtubules in metaphase [125].

As demonstrated by Ianora *et al.* [126], there is a connection between diet, spermatophore production and sperm quality: some dinoflagellate diets significantly modified spermatophore production and reduced the fertilization capacity of male copepod sperm.

#### 4.1.2. Sperm motility

As described before, mammalian spermatozoa gradually acquire flagellar motility during their passage through the epididymis [108,127,128]. Motility plays a key role in the natural fertilization process and depends on the flagellum, which contains the axoneme structure, a pair of central singlet microtubules encircled by nine outer doublet microtubules [129]. Dynein, found in the doublet microtubule arms, has ATPase activity and plays a role energizing flagellar and ciliary movement. It has now been established that the fundamental mechanism underlying flagellar and ciliary movement is the sliding between the adjacent outer doublet microtubules, which is mediated by the dynein arms [130,131].

Gelsolin drastically inhibited guinea pig sperm motility in a dose dependent manner, while, on the contrary, **LAT** did not affect it. Since gelsolin is a specific F-actin severing protein, while **LAT** seems to associate only with actin monomers thereby preventing them from repolymerizing into filaments [26], specific intact F-actin regions play a role in supporting sperm motility [131].

There is compelling evidence of pronounced effects of **PUA** on sperm motility [64]. **DD** inhibits motility in a clear dose- and time-dependent manner for a range of broadcast spawning marine invertebrates, but importantly does not result in sperm death. Using the broadcast-spawning echinoderms *Asterias rubens* and *Psammechinus miliaris* and the polychaetes *Arenicola marina* and *Nereis virens* as model species, Caldwell *et al.* [64] demonstrated the inhibitory effect of **DD** on sperm motility. When sperm were incubated with **DD**, the effects on fertilization success with untreated oocytes were striking and a pronounced reduction in fertilization success was observed. Although flagellar beating was arrested, the sperm remained viable, as evidenced by prolonged oscillation of the sperm head. Beyond the **PUA**-mediated cytoskeletal disruption, it is likely that the motility inhibition is also due to interference with calcium signaling, since it has been demonstrated that **DD** inhibits the fertilization current in ascidians together with the voltage-gated calcium current activity [132] and that calcium ions play an essential role in the normal functioning of marine invertebrate sperm, greatly influencing the pattern and shape of flagellar bending [133]. In fact, in sea urchin sperm, elevated calcium increases flagellar beat asymmetry and reversibly blocks beating [134–136]. In mammalian sperm, the progressive decrease of internal calcium concentration during epididymal maturation is essential to prepare the sperm for activation of motility whereas internal calcium concentration elevation is required at ejaculation to develop flagellar motion [137]. Beyond actin cytoskeleton modifications, possibly also sperm motility inhibition by **PAL** could be explained by toxin-associated cytosolic calcium changes [61]. The motility of hamster caudal epididymal and other sperm can be inhibited by **PAL** in a time-dependent manner, detectable as a loss in flagellar amplitude, often accompanied by an increase in beat frequency, resulting in a loss of forward progression and ultimately cessation of movement. Similar effects were observed in sperm from guinea pigs, rabbit, cattle, sea urchins and human. The observation showing **PAL** did not inhibit the progressive motility of demembranated sperm axoneme preparations suggests that this large molecule acts *via* the plasma membrane to cause its exceedingly toxic effects [138].

#### 4.1.3. Oogenesis

During oogenesis, the immature oocyte grows in size and acquires the competence to mature and then to be fertilized. Oocyte maturation is the last phase of oogenesis and consists of nuclear and cytoplasmic modifications [139,140]. Nuclear maturation involves a cell cycle progression, which alternates between meiotic arrest and resumption; the first meiotic arrest occurs during prophase I, when the immature oocyte is characterized by a large nucleus known as the germinal vesicle (GV). As a general scheme, the first morphological indication of nuclear maturation is represented by germinal vesicle breakdown (GVBD), which, depending on the species, is induced by a different stimulus; in the majority of species, the oocyte subsequently arrests at first (MI) or second metaphase (MII) stage, where it remains up to the time of fertilization or parthenogenic activation [141]. The meiotic stage correlated with fertilizable oocyte is species-specific: in some animals, oocytes are fertilized at the GV stage (e.g., some molluscs) or, on the contrary, there are some oocytes that are fertilized after meiosis completion (coelenterate, echinoderms). The second oocyte arrest occurs at MI stage in worms, ascidians, and some molluscs, or at MII stage in the *Amphioxus* and in all the vertebrates [141].

The process of cytoplasmic maturation is less clear, but is characterized by morphological and functional changes that are necessary to support fertilization and the subsequent developmental events [142–144]. These changes include modification of the plasma membrane, calcium signaling, and activation of specific molecules and complexes [145–150].

In *Ciona intestinalis*, oocyte growth is associated with characteristic changes in the distribution of mitochondria, microtubules and cortical mRNAs, which all translocate from the region surrounding the GV in previtellogenic and vitellogenic oocytes to the periphery of the postvitellogenic oocytes [151]. Similar types of relocalizations occur in the *Xenopus* previtellogenic oocyte in which microtubular reorganizations and the translocation of mRNAs to the cortex have been well studied [152,153].

It has been reported that redistribution of endoplasmic reticulum in the cytoplasm to the nuclear area is dependent on microfilaments in starfish eggs [154] and on microtubules in *Drosophila* [155]. During mammalian oocyte growth, organelles move to the cell cortex, forming an ‘organelle zone’, while organelles (except for cortical granules) move centrally during oocyte maturation, forming an ‘organelle-free zone’ at the cortex of a mature oocyte. Mitochondrial translocation is mediated by microtubules, not by microfilaments, since disruption of microtubules, but not microfilaments, blocked mitochondria migration [156]. Centrosomes, other organelles, govern the organization of microtubules: their separation is regulated by microfilaments in sea urchin [157] and mouse [158] eggs, while microtubule inhibition prevents centrosome expansion and separation in sea urchin [157]. In sea urchin [159], pig [156], and mouse [160], cortical granule translocation to the egg cortex is driven by microfilaments. In mammalian oocytes, many of the maturation and fertilization events are driven by the dynamic interactions between myosin and actin filaments whose polymerization is regulated by RhoA, Cdc42, Arp2/3 and other signaling molecules [11,161].

In *Ciona*, as in some molluscs and mammals, the reinitiation of meiosis causes GVBD and formation of a central meiotic spindle which migrates towards the oocyte surface [151,162]. In contrast, in starfish and amphibian oocytes, the GV is located eccentrically just beneath the cortex and defines the future position of the meiotic spindle [163,164]. Finally, in some species of sea urchins, sea cucumbers, and fishes meiosis resumption triggers GV migration towards the surface and subsequent GVBD and meiotic spindle formation at that site [165,166].

Mechanisms involved in the migration and positioning of the meiotic spindle have been examined in a few species, and there are examples of both microtubule-dependent and actin-dependent movements. In oocytes of starfish and sea cucumbers, microtubules and centrosomes participate in GV relocation in an eccentric position just beneath the oocyte cortex [163,165]. In *C. elegans*, the translocation of the meiotic spindle to the oocyte cortex appears to be mediated by a microtubule-associated kinesin [167]. Movement of the meiotic spindle toward the unique cortical site is microtubule-dependent also in oocytes of the worm *Chaetopterus* [168]. In contrast, in *Xenopus* oocytes, a myosin (Myo10) plays a critical role in GV anchoring, meiotic spindle assembly and anchoring to the cortex by integrating the actin microfilament and microtubule cytoskeletons [169]. In mouse oocytes, a large meiotic spindle forms in the center of the oocyte after GVBD and migrates towards the region of the cortex closest to one pole of the spindle in an actin-dependent manner [170]. Infact, this spindle migration to the oocyte cortex is prevented by **JAS**, which induces microfilament polymerization and stabilization [171].

GVBD and meiotic spindle formation are not regulated by microfilaments, but polarized movement of the chromosomes depends on a microfilament-mediated process in maturing mouse oocytes. Similar results have been obtained in other species, such as hamster [172], cattle [173], pig [156] and human [174], while in *Xenopus* the results are contradictory [11,175,176].

In maturing oocytes of *C. intestinalis*, the first visible sign of polarization is the microfilament-dependent migration of the meiotic apparatus toward the oocyte surface. After the localization of the meiotic apparatus under the animal pole, the myoplasm, the mitochondria-rich domain featured in all species of ascidians, polarizes along the animal/vegetal axis, and this polarization is blocked by microfilament inhibitors but not by microtubule inhibitors. In the same ascidian species, experiments with non-derived marine drugs (cytochalasin, affecting actin cytoskeleton) indicate that actin is responsible for the transition of the myoplasm from a uniform layer of mitochondria to a polarized basket lining the equatorial and vegetal regions [151].

As described above, Caldwell [64] found that **DD** affected sperm motility, while pre-incubation of oocytes in **DD** affected fertilization success to a limited degree, so influencing fertilization success from the paternal side. However, maternal effects cannot be discounted. Poulet *et al.* [177] have formulated the hypothesis that, following diatom-feeding, toxins are incorporated into oocytes during oogenesis so limiting the fertilization success. When maturation was initiated in the presence of **DD**, the oocytes underwent a severe cellular disruptive event. **DD** therefore is cytotoxic during the prophase/metaphase transition and may have an important role in determining oocyte viability in diatom-feeding invertebrates. It is more feasible that low level molecular and cellular damage is occurring such as DNA damage, and where the rate of damage exceeds the capacity of the adult to repair the damage, reproductive effects may be observed. Such damage is particularly relevant for oocyte maturation [63].

In starfish, the two most immediate responses to the maturation inducing hormone 1-methyladenine are the quick release of intracellular calcium and the accelerated changes of the actin cytoskeleton in the cortex. The finding that **JAS** inhibited the 1-methyladenine -induced calcium response suggests that the dynamic change of actin cytoskeleton may play a regulatory role in modulating intracellular calcium release [178].

In starfish *Asterinia pectinifiera*, **TEO** induced malformation of immature oocytes through disturbance of cortical F-actin distribution. Instead, no morphological changes were observed in maturing oocytes when the same drug was added to oocytes which had been induced to mature by 1-methyladenine [51]. In the same starfish species, the oocyte maturation was found to be arrested by ten different **STR** by affecting actin dynamics [179].

It has been demonstrated that **OA** induces GVBD in starfishes [180,181] and frogs [182]. In mammals where, as stated before, in contrast to other zoological groups, oocyte maturation takes place spontaneously, **OA** has been shown to induce chromatin condensation and GVBD in mouse oocytes arrested at GV stage by maturation inhibitor [89,183–186]. Although continuous exposure of macaque oocytes to **CLA** enhances GVBD and results in oocytes displaying various cytoplasmic aberrations, transient treatment with **CLA** or **OA** stimulates GVBD without increasing the incidence of morphological abnormalities. These transiently treated oocytes retain the ability to develop to MII and to fertilize [46]. However, it is important to bear in mind that **OA** alone might exert pleiotropic effects on oocyte as a consequence of the multiplicity of its molecular targets [89].

### 4.2. Gamete Activation and Fertilization

Fertilization is a highly specialized process of cell-cell interaction that marks the creation of a new and unique individual. It is a complex multi-step process involving many events, including gamete recognition, binding, activation, and fusion. Reciprocal activation of the two gametes is a crucial step of these events; signals from the oocyte investments induce dramatic changes in form and function of the spermatozoon, and the spermatozoon triggers the quiescent oocyte into metabolic activation (for review see [108]).

#### 4.2.1. Sperm capacitation and acrosome reaction

In order to fertilize, the mammalian spermatozoa should reside in the female reproductive tract for several hours, during which they undergo a series of biochemical modifications collectively called capacitation. Only capacitated spermatozoa can undergo the acrosome reaction (AR) after binding to the egg zona pellucida, a process which enables sperm to penetrate into the egg and fertilize it [187]. Polymerization of G-actin to F-actin occurs during capacitation. It was reported that actin polymerization is important for initiation of sperm motility during post-testicular maturation [188]. The location of actin in the acrosomal region of several mammalian species [189–194] supports its possible role in sperm capacitation and the acrosome reaction. Actin polymerization is necessary for sperm incorporation into the egg cytoplasm [195] and for sperm nuclei decondensation [196]. Sperm from sea urchins, but not those from Limulus or mice, were affected by **LAT**, becoming unable to assemble acrosomal processes and their ability to fertilize eggs is impaired [197].

The AR is the last activating event in the spermatozoon as it becomes competent for fertilization. The exocytosis of the acrosome and the consequent release of its contained enzymes allows the spermatozoon to penetrate the extracellular oocyte investments [198]. Prior to the occurrence of the acrosome reaction, the F-actin should undergo depolymerization, a necessary process which enables the outer acrosomal membrane and the overlying plasma membrane to come into close proximity and fuse. The binding of the capacitated sperm to the zona pellucida induces a fast increase in sperm intracellular calcium, activation of actin severing proteins which break down the actin fibers, and allows the acrosome reaction to take place [187].

#### 4.2.2. Fertilization

In mammals, sperm-oocyte fusion occurs predominantly [199,200] or exclusively [201] in the microvillar-rich region of egg surface. In mollusc and echinoderm, a fertilizing sperm contacting the egg protrudes the acrosomal vacuole by elongating the central axial actin bundles [202,203]. Shortly after fusion, a global exocytic event occurs in the oocyte and the contents of cortical secretory granules are released into the extracellular milieu. Enzymes included in these granules modify the zona pellucida in a way that prevents further sperm penetration through this matrix [204]. The polymerization of actin beneath the plasma membrane of the fertilization cone and inhibition of sperm incorporation by cytochalasins or **LAT A** are observed in eggs of zebrafish and sea urchin [197,205,206].

In the red alga *Bostrychia moritziana*, the actin inhibitors **JAS**, **MYC**, **LAT A** and **B** inhibited gamete fusion affecting the formation of fertilization pore [207].

In starfish eggs, subplasmalemmal actin fibers are involved in the process of sperm-egg interaction and in the subsequent events related to fertilization: the alteration of the cortical actin networks by the use of **LAT A** or **JAS** led to the deregulation of monospermic sperm interaction, generation of calcium signaling, of cortical granule exocytosis, and of the sperm entry process [208]. The regulation of actin polymerization is also involved in the membrane block to polyspermy in mouse eggs [209]. In contrast, microtubule polymerization was apparently unnecessary for this initial process of sperm entry, as demonstrated in mussel [210]. Unfortunately, the need of microfilaments for sperm incorporation is somewhat inconclusive in mammals [11] and thus requires further investigation.

Oocyte activation is a dynamic mechanism, and its progress is characterized by early events, e.g., cortical granule exocytosis and fertilization currents, and late events, such as resumption of meiosis [211,212]. In mouse **JAS** was found to prevent cortical granule exocytosis after artificial activation [171], suggesting that actin filaments participate in cortical granule exocytosis. In contrast, neither microfilaments nor microtubules are involved in this event during pig oocyte activation [156], since egg exposition either to cytochalasin B or to marine drug **JAS** did not prevent cortical granule exocytosis [11]. In support of this hypothesis, the initial cortical release of calcium promoted by sperm may be due to depolymerization of actin in starfish [213].

Unlike other species, ascidian oocytes lack cortical granules, and between fertilization and first cleavage there are two major phases of reorganization corresponding to the so-called first and second phase of “ooplasmic segregation” [214]. Among consequences of sperm-egg fusion there is a calcium wave, which starts from the site of fertilization and traverse the egg [215–217]. The rise in free calcium triggers a microfilament-dependent cortical contraction wave starting on the side of the egg where fertilization occurs [216,218]. The large reorganization of the oocyte caused by the fertilization/contraction phase has traditionally been called “the first phase of ooplasmic segregation” [216,219,220]. The “second major phase of reorganization” is characterized by the events from meiosis completion to first mitotic division. The completion of the meiotic cell cycle results in the formation of male and female pronuclei around the chromosomes and the growth of a large microtubular sperm aster in the future posterior pole. As the female pronucleus starts migrating along astral microtubules toward the duplicated sperm aster and male pronucleus, these latter structures move away from the cortex and toward the center of the oocyte. These translocations during the second phase of reorganization are driven by microtubules. Once the male pronucleus and female pronucleus are in the center of the zygote, but before the first cleavage, a general surface movement occurs, which depends on actin microfilaments, necessary to complete the translocation of the myoplasm and the endoplamsic reticulum domain toward the posterior region [166]. In the ascidian *C. intestinalis*, it has been demonstrated that **DD** and **decatrienal** inhibit actin reorganization, as well as the fertilization current and voltage-gated calcium activity of the plasma membrane [132].

The contractile ring is a network of actin and myosin filaments, and the motor activity of myosin translocates actin filaments to drive its constriction [221]. In mitotic cells, contractile ring assembly is directed by the RhoA guanosine triphosphatase (GTPase), which activates myosin and actin filament assembly [221]. Accumulating evidence shows that oocytes adopt similar mechanisms for releasing the first and second polar body during maturation and fertilization. In mouse oocytes, inhibition of RhoA caused abnormal microfilament organization and blocked first and second polar body extrusion [222]. When actin filaments are disrupted, polar body emission is blocked in mouse [223], hamster [172] and sheep [224]. In mice, **JAS** prevents egg polar body emission at a much lower concentration than either cytochalasin B or marine drug **LAT A** [171].

Caldwell *et al.* [225] have demonstrated that both crude diatom extracts and **DD** can, in a dose-dependent manner, inhibit secondary fertilization processes (e.g., cortical reaction) in marine invertebrates. Also, Buttino *et al.* [226] have observed that sea urchin embryos incubated with extracts of *Thalassiosira rotula* shortly after sperm/egg binding were unable to complete pronuclear fusion due to depolymerization of microtubule assemblies.

During fertilization in sea urchin, porcine, bovine and human fertilized eggs, the sperm introduces the centrosome into the egg, and microtubules nucleated by centrosomes cause the union of male and female pronuclei [156,227–229]. Both microtubules and microfilaments are required for pronuclear apposition in the mouse [171,205]. The migrations of the sperm and egg nuclei during sea urchin fertilization are dependent on microtubules organized into a radial monastral array, the sperm aster [230].

### 4.3. Early Development

Successful fertilization drives the oocyte into meiosis completion and exit leading to formation of the zygote. This represents the first diploid cell of a new organism that divides by mitosis into a number of smaller cells named blastomeres. This process is called cleavage that differs depending on the species [141].

In fertilized eggs of sea urchin and sand dollar, accumulation of the contractile ring microfilaments at the equatorial cell cortex was first noticed at the beginning of telophase (shortly before furrow formation), and the accumulated microfilaments were organized into parallel bundles as furrowing progressed [231]. In *Xenopus* eggs, actin filament patches grow rapidly in the furrow region and, together with myosin II, form the contractile ring [232,233]. Microtubules also have an essential role in controlling the formation of the contractile ring in *Xenopus* and in mammals: in *Xenopus* eggs, microtubules are involved in advancement of the cleavage furrow [234], while in mammalian somatic cells the complete disassembly of microtubules in anaphase results in the inability to accumulate actin to form a contractile ring [235]. During cytokinesis of the mouse egg, actin involvement is demonstrated by experiments where microfilament inhibitors block cleavage [197,230], and also RhoA plays essential roles in the formation of the actin filaments and the cleavage furrow [222]. A microfilament-rich cleavage furrow was also observed in fertilized pig eggs, in which actin filaments are required for cleavage [229].

The inhibitory activities of unsaturated short chain aldehydes have been demonstrated in copepod, polychaete, echinoderm and ascidian embryos [132,225,236–238]. Effects of these bioactive compounds derived from diatoms include the arrest of embryogenesis, induction of teratogenic effects in larvae and inhibition of fertilization success. In particular, **DD** altered actin filaments, tubulin polymerization, DNA replication and mitochondrial migration after contraction, leading to a disturbance in cleavage formation. However, **DD** also induced larval teratogeny at low concentrations, possibly due to actin perturbation. Interestingly, this same **DD** concentration failed to inhibit fertilization currents and voltage gated calcium channels [132].

**LAT A** showed to be a potent inhibitor of microfilament-mediated processes, even those occurring after fertilization, because it inhibits second polar body formation and cytokinesis in mouse fertilized eggs [197].

**PSE** inhibited cytokinesis and induced formation of multinucleate cells in fertilized *Strongylocentrotus purpuratus* embryos. This toxin inhibited cytokinesis selectively by disrupting the contractile ring, whereas spindle microtubule organization and mitotic chromosome segregation to opposite spindle poles were unimpaired. The effects of **PSE** in fertilized sea urchin embryos were strikingly similar to those of another marine natural product, **STY;** both toxins could have the same cellular target(s) such as an especially sensitive sulfhydryl containing protein(s) involved in the formation or function of the contractile ring [239].

Other peptides, **GEO**, also inhibited the first cleavage of sea urchin eggs (*Lytechinus variegatus*): duplication of nuclei without complete cell division indicated that the mechanism of action might be related to microfilament disruption [47].

Microinjection of purified **AZA-1** in Japanese medaka (*Oryzias latipes*) embryo demonstrated that **AZA-1** is a potent teratogen to finfish, caused dose-dependent effects on developmental rate, hatching success, and viability in medaka embryos [240].

Using a novel oocyte-based screening system, Chae and colleagues [241] identified natural compounds that inhibit cytokinesis: when treated with **PTX-2**, mammalian ovulated oocytes activated by ethanol failed to complete mitotic division and remained in the one-cell stage.

Among marine drugs affecting microtubules, **CYN**, **DOL 15** and **MET** have been shown to alter or inhibit the egg cleavage. **CYN** blocked cleavage of developing sea urchin eggs [92,242,243], most likely because it inhibits microtubule polymerization [98]. **DOL 15** induced cleavage alteration/arrest of sea urchin fertilized eggs [244]. **MET** inhibited the cleavages of sea urchin *Sphaerechinus granularis* and *Paracentrotus lividus* fertilized eggs. The treatment severely disturbs the establishment of a mitotic spindle, most likely by affecting microtubule dynamics [104].

Table 1. summarizes the effects of marine toxins targeting cytoskeleton on different reproductive events.

## 5. Conclusions

To date, over 16,000 compounds have been isolated from marine organisms, and new compounds are continuously being discovered [245]. Discoveries of these marine natural products have been reported in approximately 7,000 publications. In addition, there are another 9,000 publications on the subject of marine natural products which deal with the syntheses, reviews, biological activity studies, ecological studies, *etc.* Furthermore, over 300 patents have been issued on bioactive marine natural products [245–248]. Some of these products may find important applications in biomedical research, agriculture, aquaculture, and chemical industries (see [249] for a review), with particular interest towards the discovery and the development of novel antitumor and cytotoxic compounds [48].

In this review we have reported many marine toxins with impact on reproductive processes by targeting microtubules or microfilaments or by affecting cytoskeletal dynamics. The effect of these marine toxins on reproductive and developmental processes is extensive and variable, affecting every step of these processes from the invertebrates up to the vertebrates, including humans. The range of reproductive impacts includes: sperm maturation, sperm motility, oocyte maturation, fertilization, and early development. The use of marine drugs could lead to many new applications such as the development of novel contraceptive methods. Therefore, one of the most important targets of pharmacology is now to screen the huge potential of marine toxins and select those that display a specific mode of action with the desired characteristics against a disease. Moreover, the study of these substances could help in the control of harmful species and lead to the improvement of protection of public health and natural environment, or to a better yield in aquaculture and agriculture field. This is a further confirmation of the importance in studying the promising and almost unexplored world of marine drugs. In conclusion, data described in the review support the fact that sperm, egg and embryo bioassays represent a promising field in screening for new drugs that affect reproductive events.

## Figures and Tables

**Table 1:** The effects of marine drugs on cytoskeleton-mediated reproductive events.

Drug Name	Drug Source	Cellular Target	Stage/Event Affected	References
Azaspiracid	Dinoflagellates	Unknown	Early development	[240]
Calyculin	Sponges	Protein phosphatases	Oogenesis	[46]
Caulerpenyne	Algae	Microtubules	Early development	[92,242,243]
2E,4E-Decadienal	Diatoms	Cytoskeleton, calcium signaling *etc.*	Sperm motility, oogenesis, fertilization, early development	[64,132,177,225–238]
Dolastatin	Molluscs	Microtubules	Early development	[244]
Geodiamolide	Sponges	Microfilaments	Early development	[47]
Jasplakinolide	Sponges	Microfilaments	Oogenesis	[171,178,207,208]
Latrunculin	Sponges	Microfilaments	Spermatogenesis, acrosome reaction, fertilization, early development	[125,197, 205–208]
Methoxyconidiol	Ascidians	Unknown	Early development	[104]
Mycalolide	Sponges	Microfilaments	Fertilization	[207]
Okadaic acid	Dinoflagellates	Protein phosphatases	Oogenesis	[89,180–186]
Palytoxin	Dinoflagellates?	Sodium pump	Sperm motility	[138]
Pectenotoxin	Dinoflagellates	Microfilaments	Early development	[241]
Pseudopterolide	Soft corals	Unknown	Early development	[239]
Strongylophorine	Sponges	Rho-GTPases	Oogenesis	[179]
Stypoldione	Algae	Microtubules (sulfhydryl groups)	Early development	[239]
Swinholide	Sponges	Microfilaments	Spermatogenesis	[125]
Theonellapeptolide	Sponges	Microfilaments	Oogenesis	[51]

## Abbreviations

(AZA): Azaspiracid
(CLA): Calyculin-A
(CYN): Caulerpenyne
(DD): 2E, 4E-Decadienal
(DOL): Dolastatin
(GEO): Geodiamolide
(JAS): Jasplakinolide
(LAT): Latrunculin
(MET): Methoxyconidiol
(MYC): Mycalolide-B
(OA): Okadaic acid
(PAL): Palytoxin
(PTX): Pectenotoxin
(PSE): Pseudopterolide
(STR): Strongylophorine
(STY): Stypoldione
(SWI): Swinholide-A
(TEO): Theonellapeptolide Ie
